# Probing the Environment of Emerin by Enhanced Ascorbate Peroxidase 2 (APEX2)-Mediated Proximity Labeling

**DOI:** 10.3390/cells9030605

**Published:** 2020-03-03

**Authors:** Marret Müller, Christina James, Christof Lenz, Henning Urlaub, Ralph H. Kehlenbach

**Affiliations:** 1 Department of Molecular Biology, Faculty of Medicine, GZMB, Georg-August-University Göttingen, Humboldtallee 23, 37073 Göttingen, Germany; marret.mueller@gmx.de (M.M.); christina.james@med.uni-goettingen.de (C.J.); 2 Bioanalytics Group, Institute of Clinical Chemistry, University Medical Center Göttingen, Robert-Koch-Straße 40, 37075 Göttingen, Germany; christof.lenz@med.uni-goettingen.de (C.L.); henning.urlaub@mpibpc.mpg.de (H.U.); 3 Bioanalytical Mass Spectrometry Group, Max Planck Institute for Biophysical Chemistry, Am Fassberg 11, 37077 Göttingen, Germany

**Keywords:** proximity labeling, APEX, RAPIDS, emerin, inner nuclear membrane

## Abstract

Emerin is one of the best characterized proteins of the inner nuclear membrane, but can also occur at the level of the endoplasmic reticulum. We now use enhanced ascorbate peroxidase 2 (APEX2) to probe the environment of emerin. APEX2 can be used as a genetic tag that produces short-lived yet highly reactive biotin species, allowing the modification of proteins that interact with or are in very close proximity to the tagged protein. Biotinylated proteins can be isolated using immobilized streptavidin and analyzed by mass spectrometry. As an alternative to the standard approach with a genetic fusion of APEX2 to emerin, we also used RAPIDS (rapamycin- and APEX-dependent identification of proteins by SILAC), a method with improved specificity, where the peroxidase interacts with the protein of interest (i.e., emerin) only upon addition of rapamycin to the cells. We compare these different approaches, which, together, identify well-known interaction partners of emerin like lamin A and the lamina associated polypeptide 1 (LAP1), as well as novel proximity partners.

## 1. Introduction

The nuclear envelope (NE) separates the genetic material in the nucleus from the cytoplasm and all its organelles. It comprises the outer nuclear membrane (ONM), which is continuous with the membrane system of the endoplasmic reticulum (ER), the inner nuclear membrane (INM) and the nuclear pore complexes (NPC), where INM and ONM are connected. In metazoans, a nuclear lamina is associated with the nuclear face of the NE, with characteristic intermediate filament proteins (lamins) tightly binding to INM-proteins. The protein composition of the INM is clearly distinct from that of the ONM and in recent years a large number of INM-specific proteins have been identified [1,2,3]. Emerin, loss of which causes X-linked Emery-Dreifuss muscular dystrophy [4,5,6,7,8], is one of the best-characterized proteins of the INM [9,10,11], although it was also described as a component of the ONM and the peripheral ER [12]. It is a tail-anchored protein with a single transmembrane domain close to its C-terminal end and we recently showed that emerin is inserted post-translationally into ER-membranes via the GET/TRC40 pathway [13]. Emerin has an N-terminal LEM-domain, which received its name from three proteins containing this characteristic feature (Lap2, emerin and Man1; [14]). Major binding partners of emerin are A- and also B-type lamins [6]. Indeed, the localization of emerin at the nuclear envelope was shown to depend on lamin A [15]. Hence, retention of emerin upon binding to lamins plays an important role in INM-targeting. Besides lamins, a number of emerin interaction partners have been described, for example BAF (barrier-to-autointegration factor (BANF1) [16], nesprin-1α [17] and HDAC3 [18]; for review see [9]). 

For proteins of the INM, the identification of interacting proteins is particularly challenging, because conditions that are typically used in, for example, co-immunoprecipitation approaches, may not lead to complete solubilization of NE-structures. Genetic approaches like the yeast-two-hybrid method, on the other hand, detect interactions under rather non-physiological conditions. Neither approach addresses the native subcellular localization of a protein of interest. To overcome these problems, several methods have been developed that use proximity-based labeling, typically introducing biotin into unknown proteins, allowing subsequent affinity capture with immobilized streptavidin and analysis by mass-spectrometry (for review see [19]). One approach termed BioID uses a biotin ligase from *E. coli* for biotinylation of proteins [20]. As an alternative, ascorbate peroxidase (APEX), an enzyme that generates radicals from biotin phenol in the presence of H_2_O_2_ was introduced [21]. Modification of proteins occurs within a range of ~20 nm of the enzyme, which can be genetically fused to the N- or C-terminus of proteins of interest for probing their direct environment and identifying potential interaction partners [22,23,24,25]. An improved version of APEX, APEX2, is far more active than the original enzyme [26]. Very recently, we devised a method where APEX2 is not directly fused, but instead targeted to a protein of interest, in a rapamycin-dependent manner [27]. The method utilizes FKBP12-tagged APEX2, which interacts with an FRB-tagged protein of interest upon addition of rapamycin to intact HeLa cells for a short period of time. SILAC, followed by quantitative mass spectrometry, then allows the comparison of proteins that get biotinylated by APEX2 in either the absence or the presence of rapamycin. Using this method (Rapamycin- and APEX-dependent identification of proteins by SILAC; RAPIDS), we identified lamin A and several other proteins that directly interact with or are in close proximity to emerin.

## 2. Materials and Methods

### 2.1. Plasmids

All plasmids were cloned using standard procedures and were verified by sequencing. Plasmids coding for APEX2-dGFP-NLS-FKBP12 and FKBP12-GFP-APEX2 were described before [27]. pcDNA3-FKBP12-GFP-APEX2 was used as a template to amplify the APEX2 coding sequence by using primers G1852 (5′-GCGCTAGCGTCGCCACCATGGGAAAGTCTTACCCAACTGTGAG) and G1853 (5′-GCCTCGAGAACCAGAAGCTCCTGAGGCATCAGCAAACCCAAGCTCG) or G1854 (5′-GCGAATTCTGGAAAGTCTTACCCAACTGTGAG) and G1855 (5′-GCGGATCCGGCATCAGCAAACCCAAGCTC). The PCR products were cloned into a pmCherry-C1 derivative, encoding mCherry-emerin and into pEGFP-C1 (Clontech Laboratories, Mountain View, CA, USA), generating pAPEX2-emerin and pGFP-APEX2, respectively. Cloning of pmCherry-FRB-emerin was described previously [13]. All fusion proteins contained the full-length sequence of emerin or APEX2, respectively, with short linker sequences as determined by the restriction sites.

### 2.2. Cell Culture and Transfection

HeLa P4 cells [28] were obtained from the NIH AIDS Reagent Program and grown in DMEM (Life technologies, Carlsbad, CA, USA) supplemented with 10% (v/v) FBS (Life technologies, Carlsbad, CA, USA), 100 U mL^−1^ penicillin, 100 µg mL^−1^ streptomycin and 2 mM L-glutamine (Life technologies, Carlsbad, CA, USA) under 5% CO_2_ at 37°C. Cells were tested for mycoplasma contamination on a regular basis.

For SILAC, HeLa cells were labeled with heavy or light isotopes of arginine and lysine. For this purpose, DMEM (high glucose) lacking glutamine, lysine and arginine (Thermo Fisher Scientific, Waltham, MA, USA) was supplemented with 10% (v/v) dialyzed FBS (Life technologies, Carlsbad, CA, USA), 6 mM L-glutamine (Life technologies), 100 U mL^−1^ penicillin and 100 µg mL^−1^ streptomycin. SILAC media was prepared as described [27], using ^13^C_6_^15^N_2_-L-lysine (Silantes, Munich, Germany) and ^13^C_6_^15^N_4_-L-arginine (Silantes, Munich, Germany) or ^12^C_6_^14^N_2_-L-lysine (Sigma-Aldrich, St. Louis, MO, USA) and ^12^C_6_^14^N_4_-L-arginine (Sigma-Aldrich, St. Louis, MO, USA) as heavy or light amino acids, respectively. To ensure sufficient incorporation of heavy amino acids, cells were passaged five to seven times in SILAC medium before the biotinylation experiment. The incorporation rate was confirmed to be ≥97% by mass spectrometry.

Cells were transfected using the calcium phosphate method [27,29].

### 2.3. Rapamycin-Dependent Biotinylation Assay

Cells grown in SILAC medium were subjected to biotinylation reactions as described [27]. The biotinylation experiment using the FKBP12-GFP-APEX2 construct was performed in 10 cm dishes. The cells were transfected with pmCherry-FRB-emerin and pcDNA3-FKBP12-GFP-APEX2 and grown to confluency. Cells were then incubated for 30 min with 500 µM biotin-phenol (Iris Biotech, Marktredwitz, Germany), in the presence or absence of 200 nM rapamycin (Sigma Aldrich, St. Louis, MO, USA). Experiments were performed in both forward and reverse conditions. For forward reactions, cells grown in “light” SILAC medium were treated with rapamycin and cells grown in “heavy” SILAC medium were not. For reverse reactions, this labeling scheme was switched. After incubation with biotin-phenol and rapamycin, 1 mM H_2_O_2_ was added to initiate the biotinylation reaction at room temperature. After 1 min, the medium was aspirated and cells were washed twice with quenching buffer (5 mM Trolox, 10 mM NaN_3_, 10 mM sodium ascorbate in PBS) and once with PBS. Cells used for fluorescence microscopy were fixed immediately. 

For Western blot and SILAC analyses, cells from each dish were lysed with 1.4 mL RIPA buffer (50 mM Tris, pH 7.4, 5 mM Trolox, 0.5% (w/v) sodium deoxycholate, 150 mM NaCl, 0.1% (w/v) sodium dodecyl sulfate (SDS), 1% (v/v) Triton X-100, 1 mM phenylmethane sulfonyl fluoride (PMSF), 10 mM NaN_3_, 10 mM sodium ascorbate, 1 µg mL^−1^ aprotinin, 1 µg mL^−1^ leupeptin and 1 µg mL^−1^ pepstatin). The cell lysate was incubated for 5 min on ice and centrifuged for 10 min at 16,000 g and 4°C. The cleared cell lysate was used to enrich biotinylated proteins with Neutravidin beads (Thermo Fisher Scientific, Waltham, MA, USA). For mass spectrometry, cell lysates derived from three 10 cm dishes were pooled, the protein concentration of the cell lysates was determined using the Pierce 660 nm Protein Assay (Thermo Fisher Scientific, Waltham, MA, USA) and equal protein amounts of samples treated with or without rapamycin were mixed prior to addition to Neutravidin beads. For Western blot analyses, the samples were kept separately. For each forward or reverse experiment, six batches of 140 µl Neutravidin beads were incubated with 1.4 mL cell lysate overnight at 4 °C on a rotor. The beads were washed once with washing buffer 1 (50 mM HEPES (pH 7.4), 0.1% (w/v) sodium deoxycholate, 1% (v/v) Triton X-100, 500 mM NaCl, 1 mM ethylenediaminetetraacetic acid (EDTA), once with washing buffer 2 (50 mM Tris (pH 8.0), 250 mM LiCl, 0.5% (v/v) Nonidet P-40, 0.5% (w/v) sodium deoxycholate, 1 mM EDTA) and twice with washing buffer 3 (50 mM Tris (pH 7.4) and 50 mM NaCl). For each washing step, the beads were incubated for 8 min at 4 °C on a rotor. After the last washing step, the buffer was removed carefully and bound proteins were eluted from the beads by incubation for 5 min at 95 °C with 90 µl SDS sample buffer (4% (w/v) SDS, 125 mM Tris pH 6.8, 10% (v/v) glycerol, 0.02% (v/v) bromophenol blue, 10% (v/v) β-mercaptoethanol) supplemented with 5 mM desthiobiotin (Sigma-Aldrich, St. Louis, MO, USA). To increase the protein concentration, three batches of beads were consecutively eluted in the same buffer.

The biotinylation experiment using APEX2-dGFP-NLS-FKBP12 was performed as described above with the following changes: Cells were seeded in six-well plates and transfected with pmCherry-FRB-emerin and pAPEX2-dGFP-NLS-FKBP12. Cells of two wells were lysed in 0.5 mL RIPA buffer. In total, 6 mL of rapamycin-treated cell lysate and 6 mL of lysate derived from cells that had not been treated with rapamycin were obtained and the same protein amounts were mixed. 24 batches of 40 µL Neutravidin beads were incubated with 0.5 mL cell lysate each. For elution, three batches of beads were consecutively incubated in 40 µL SDS sample buffer.

### 2.4. Standard APEX-Biotinylation Assay

The biotinylation experiment using APEX2 directly fused to emerin was performed as described above for the FKBP12-GFP-APEX2 construct with the following changes: instead of comparing biotinylated proteins with or without incubation of cells with rapamycin, the biotinylation of proteins in cells that had been transfected with either pAPEX2-emerin or with pGFP-APEX2 was compared by SILAC.

### 2.5. Mass Spectrometric Analyses

Samples were separated on 4–12% NuPAGE Novex Bis-Tris Minigels (Invitrogen, Carlsbad, California). Gels were stained with Coomassie Blue, and each lane sliced into 11–12 equidistant bands. After washing, gel slices were reduced with dithiothreitol (DTT), alkylated with 2-iodoacetamide and digested with trypsin (sequencing grade, Promega, Madison Wisconsin) overnight. The resulting peptide mixtures were then extracted, dried in a SpeedVac, reconstituted in 2% acetonitrile/0.1% formic acid (v/v) and analyzed by nano-LC-MS/MS on a hybrid quadrupole/orbitrap mass spectrometer (Q Exactive, Thermo Fisher Scientific, Dreieich, Germany) as described previously [30]. Raw data processing and analysis was performed as described [27], using MaxQuant Software version 1.5.7.4 (Max Planck Institute for Biochemistry, Martinsried, Germany). Perseus Software version 1.5.6.0 (Max Planck Institute for Biochemistry, Martinsried, Germany) was used for statistical evaluation of relative protein quantitation values and a two-sided Significance B test [31] was performed using normalized log_2_ ratios. For the analysis, a Benjamini-Hochberg correction was applied and a threshold value of 0.05 was chosen. For the rapamycin approach, experiments were performed twice, each with two biological and two technical replicates. For the standard APEX-approach, only one experiment was performed, again with two biological and two technical replicates.

### 2.6. Data Availability

The MS proteomics data have been deposited to the ProteomeXchange Consortium via the PRIDE [32] partner repository with the dataset identifier PXD009783.

### 2.7. Western Blot Analyses

Western blotting was performed according to standard methods using HRP-coupled secondary antibodies. To detect biotinylated proteins, they were separated by SDS-PAGE using Mini Protean TGX Precast gels (4–20%; Biorad, Hercules, CA, USA). After transfer to nitrocellulose, the membranes were incubated in blocking buffer (3% BSA in TBS-T (24.8 mM Tris, pH 7.4, 137 mM NaCl, 2.7 mM KCl, 1% (v/v) Tween 20)) overnight at 4 °C. Incubation with streptavidin-HRP (Jackson ImmunoResearch Laboratories, West Grove, PA, USA; diluted 1:5,000–1:40,000 in blocking buffer) for 1 h at room temperature was followed by four washing steps with TBS-T. For detection of proteins, Immobilon Western Chemiluminescent HRP Substrate (Millipore, Burlington, MA, USA) was used.

### 2.8. Immunofluorescence and Fluorescence Microscopy

For fluorescence microscopy, cells were grown on coverslips and fixed with 3.7% (v/v) formaldehyde. Cells expressing fluorescently labeled proteins were mounted directly with MOWIOL supplemented with 1 µg mL^−1^ DAPI. For immunofluorescence, fixed cells were permeabilized with 0.5% (v/v) Triton X-100 in PBS for 5 min at room temperature and blocked with 3% (w/v) BSA in PBS for 15 min at room temperature. Staining was performed for 1 h at room temperature using appropriate primary antibodies and fluorescently labeled secondary antibodies (Table S5), which were diluted in 3% BSA in PBS. Afterwards, cells were embedded in MOWIOL-DAPI.

Microscopic analysis was performed using an LSM-510 confocal laser scanning microscope (Zeiss, Oberkochen, Germany) with a 63x LCI Plan-Neofluar 1.3 NA oil corrected objective.

### 2.9. Cross-Linking and Coimmunoprecipitation

HeLa P4 cells were washed twice with cold PBS containing 0.1 mM CaCl_2_ and 1 mM MgCl_2_ and incubated with dithiobis(succinimidyl propionate); (DSP; Thermo Scientific) at a final concentration of 1 mM in DMSO for 2 h on ice. For control reactions, DMSO alone was used. DSP was quenched by the addition of 20 mM Tris-HCl, pH 7.4, for 15 min. The cells were then washed twice with cold PBS and lysed with 1 mL of lysis buffer (0.5% sodium deoxycholate, 50 mM Tris-HCl, pH 7.4, 150 mM NaCl, 0.25% SDS, and 0.5% Triton X-100 with Complete protease inhibitor mixture (Roche Applied Science)) for 30 min on ice. To reduce viscosity, the lysate was passed through a 27-gauge×3/4-inch needle and then centrifuged at 15,000 g for 20 min at 4 °C. For immunoprecipitation of endogenous protein complexes, 3 µg of mouse anti-emerin, or IgG as a control were immobilized on 50 µL of Protein A-Sepharose 4 Fast Flow beads (GE Healthcare) for 3 h and incubated with lysates from 24 × 10^6^ cells that had or had not been subjected to cross-linking as described above. The beads were then washed four times with washing buffer (10 mM HEPES, 150 mM NaCl, 1 mM EGTA, 0.1 mM MgCl_2_, 0.1% Triton X-100, and Complete protease inhibitor mixture), and proteins were eluted with sample buffer containing 50 mM DTT.

### 2.10. Gene Ontology Analysis

For gene ontology cellular compartment (GOCC) analysis of all the significant proteins found in the three proteomic approaches, the *H. sapiens* proteome database was used as reference. Proteins were analyzed using WebGestalt (WEB-based Gene SeT AnaLysis Toolkit) with Benjamini-Hochberg multiple test adjustment with a significance level of the false discovery rate (FDR) of *p* < 0.05. The GO terms were selected for the classification of proteins only if there were more than five and less than 2000 proteins in the reference set.

## 3. Results

### 3.1. The Standard APEX2-Approach

**Figure 1:**
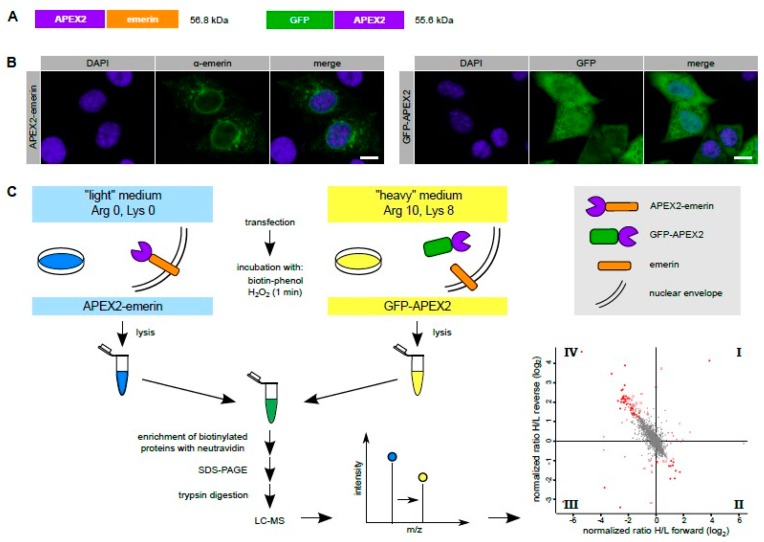
The standard APEX-approach. (**A**) Scheme of APEX2-emerin and GFP-APEX2. Note that the emerin moiety has to be at the C-terminus of the fusion protein to allow its post-translational membrane insertion. The position of the APEX2 moiety in the GFP-construct, on the other hand, is not critical. (**B**) HeLa cells were transfected with plasmids coding for APEX2-emerin or GFP-APEX2 as indicated. The subcellular localization of the overexpressed proteins was analyzed by confocal microscopy after indirect immunofluorescence using an antibody against emerin (left) or directly, detecting the GFP-signal. Bar, 10 µm. Note that under our conditions of exposure, only overexpressed APEX2-emerin but not the endogenous protein is detected. (**C**) Scheme of the experimental workflow (see text for details). The labeling scheme reflects the forward reaction (“light” medium for APEX2-emerin-transfected cells, “heavy” medium for GFP-APEX2-transfected cells). For the reverse reaction, these conditions were switched. The four-quadrant-scatter-plot shows normalized log_2_-ratios of proteins eluted from Neutravidin beads in forward and reverse experiments. Specifically biotinylated proteins are expected in quadrant IV (see Figure 2D for details).

**Figure 2:**
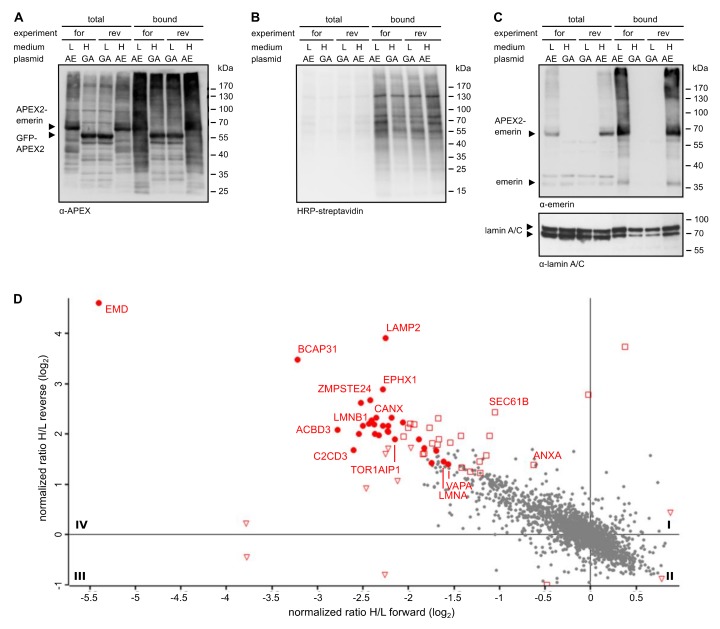
Proximity-mapping of emerin by the standard APEX-approach. Cells were transfected with plasmids coding for APEX2-emerin (AE) or GFP-APEX2 (GA), grown in “light” (L) or “heavy” (H) medium as indicated and subjected to APEX2-dependent biotinylation in forward (for) and reverse (rev) experiments. (**A**–**C**) Proteins from cell lysates were bound to Neutravidin beads and the total and the bound fractions were analyzed by immunoblotting. Anti-APEX (**A**), HRP-streptavidin (**B**) or anti-emerin and anti-lamin A/C (**C**) were used for detection. (**D**) Enlarged scatter plot of quadrant IV as also shown in Figure 1C. Closed circles: proteins significant in both experiments; open triangles: proteins significant only in forward experiments; open squares: proteins significant only in reverse experiments. Proteins of the GET/TRC40 pathway [13] were not identified as significant proximity partners.

To probe the immediate environment of emerin by a proximity-based labeling approach, we first used the standard APEX2 method and fused the peroxidase directly to emerin (Figure 1A). When overexpressed in transiently transfected HeLa cells, APEX2-emerin was found not only at its expected localization, the nuclear rim, but also outside the nucleus, probably in the ER (Figure 1B). This may in part result from overexpression effects (compare Figure 2C). On the other hand, also endogenous emerin can reside at places distinct from the INM [12]. Hence, the standard APEX2 method (and also our alternative approaches described below) may not only lead to the identification of partner proteins of emerin at the level of the INM, but also at the level of the ER. Since we expected a number of unspecific biotinylation products, we also engineered GFP-APEX2 (Figure 1A) to quantitatively compare sets of proteins that are modified in cells expressing either one of the APEX2-fusion proteins. GFP-APEX2 was detected all over the cell, including the nuclear volume (Figure 1B). For a SILAC approach (Figure 1C), cells were grown in media containing light or heavy isotopes of the amino acids lysine and arginine and transfected with plasmids coding for APEX2-emerin or GFP-APEX2, respectively.

Reactions were performed in forward and reverse mode (i.e., applying a switch of the specific labeling scheme). Transfected cells were then subjected to the biotinylation procedure. After the reaction, total proteins from cell lysates were combined and biotinylated proteins were captured using immobilized Neutravidin. Total proteins and proteins eluted from the Neutravidin beads were then analyzed by Western blotting. As shown in Figure 2A, both APEX2 fusion proteins were detected in the total cell lysates as well as in the eluted fraction, indicating similar transfection efficiencies and similar levels of self-biotinylation. When the total population of biotinylated proteins was analyzed using HRP-streptavidin as a detection reagent (Figure 2B), the pattern of proteins, which include some that are biotinylated endogenously (compare Figure S1), was rather similar for the two different fusion proteins. Next, we analyzed the blot with an antibody against emerin. In the total cell lysates, endogenous emerin as well as APEX2-emerin could be detected (Figure 2C). According to the observed intensities on the Western blot, we estimated the expression levels of APEX2-emerin to be two- to five-fold higher than those of the endogenous protein. Both proteins also eluted from the Neutravidin beads, demonstrating their biotinylation if APEX2-emerin was expressed. This finding is consistent with the previously reported self association of emerin [33]. Finally, we analyzed the blot for lamin A/C, a protein that directly interacts with emerin in the INM. As shown in Figure 2C (bottom), lamin A/C was detected in the total cell lysates and also in Neutravidin-eluates derived from cells that had been transfected with GFP-APEX2, indicating unspecific biotinylation by the soluble form of the peroxidase.

Higher levels of lamin A/C, however, were seen when the transfected cells expressed our membrane protein of interest, APEX2-emerin. Of note, slightly higher levels of lamin C than lamin A were detected in the total fraction, whereas this ratio was reversed in the bound fraction. This may indicate a preferred interaction of emerin with lamin A, but may also result from differences in the availability of biotinylation sites in lamin A and lamin C.

These results suggest that APEX2 fused to emerin can promote biotinylation of the interaction partner lamin A/C. However, background biotinylation of lamin A/C and probably other neighbouring proteins of emerin, as observed in cells expressing soluble APEX2 fused to GFP (Figure 2C), might impede a faithful identification of bona fide emerin proximity partners. Nevertheless, we decided to subject the samples eluted from the Neutravidin beads to quantitative mass spectrometry. Since the cells that expressed either APEX2-emerin or GFP-APEX2 had initially been grown in media containing either heavy or light amino acids, a comparison of heavy and light tryptic fragments of biotinylated proteins should allow the identification of proteins that were in close proximity to either fusion protein during the H_2_O_2_-pulse. In this approach, we consider proteins that are biotinylated in the presence of GFP-APEX2 as background, since this fusion protein is not expected to specifically interact with cellular proteins. Figure 1C (bottom right) and Figure 2D show the result of our “standard” APEX2 experiment, depicting proteins that were preferentially biotinylated in cells expressing APEX2-emerin as compared to cells expressing GFP-APEX2. Despite the rather small differences in biotinylation levels observed by Western blotting, several proteins with a significant score were identified as emerin-proximal proteins (see also Table S1), including the known interaction partner lamin A. Such proteins are expected in quadrant IV (enlarged in Figure 2D), where biotinylated proteins appear that are enriched in APEX2-emerin- compared to GFP-APEX2-expressing cells in both forward and reverse reactions.

### 3.2. Rapamycin- and APEX-Dependent Identification of Proteins by SILAC

In light of the rather high background of biotinylated proteins observed in cells expressing GFP-APEX2 (e.g., lamins in Figure 2C), we decided to apply RAPIDS (Rapamycin- and APEX-dependent identification of proteins by SILAC), a recently established system for APEX2-dependent identification of proteins [27].

**Figure 3:**
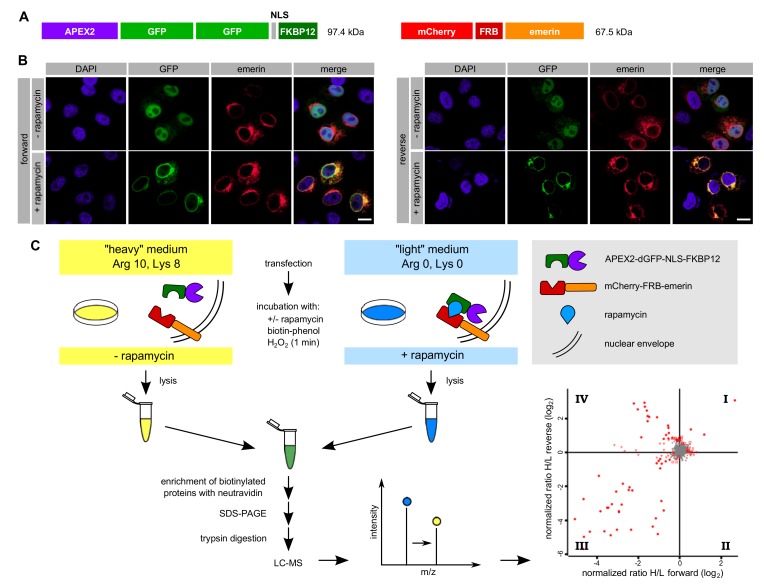
Proximity mapping of emerin by RAPIDS. (**A**) Scheme of APEX2-dGFP-NLS-FKBP12 and mCherry-FRB-emerin. (**B**,**C**) Cells were co-transfected with plasmids coding for APEX2-dGFP-NLS-FKBP12 and mCherry-FRB-emerin, grown in “light” or “heavy” medium and treated with or without rapamycin. (**B**) Cells were analyzed by confocal microscopy, detecting the GFP-signal of APEX2-dGFP-NLS-FKBP12 and the mCherry-signal of mCherry-FRB-emerin. Bars, 10 µm. (**C**) Scheme of the experimental workflow (see text for details). The labeling scheme reflects the forward reaction (“heavy” medium for cells not treated with rapamycin, “light” medium for cells treated with rapamycin). For the reverse reaction, these conditions were switched. The four-quadrant-scatter-plot resulting from two independent experiments (each with forward and reverse conditions) shows normalized log_2_-ratios of proteins eluted from Neutravidin beads. Specifically biotinylated proteins are expected in quadrant IV (see Figure 4D for details).

**Figure 4:**
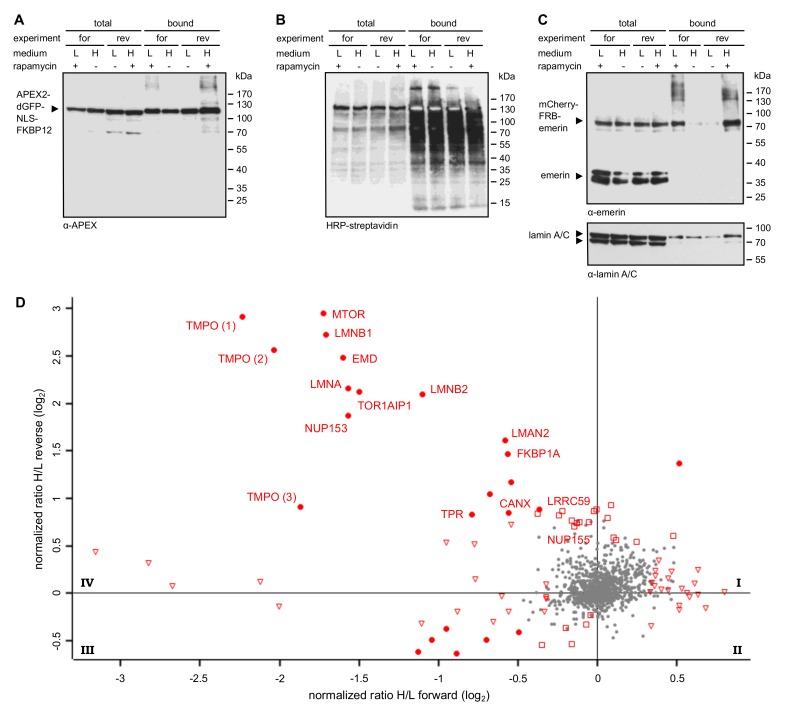
Identification of proteins interacting with emerin by RAPIDS. Cells were transfected with plasmids coding for APEX2-dGFP-NLS-FKBP12 and mCherry-FRB-emerin, grown in “light” (L) or “heavy” (H) medium as indicated and subjected to APEX2-dependent biotinylation in forward (for) and reverse (rev) experiments. (**A**–**C**) Proteins from cell lysates were bound to Neutravidin beads and the total and the bound fractions were analyzed by immunoblotting. Anti-APEX (**A**), HRP-streptavidin (**B**) or anti-emerin and anti-lamin A/C (**C**) were used for detection. Note that the levels of mCherry-FRB-emerin were much lower than those of the endogenous protein. (**D**) Enlarged scatter plot of quadrant IV as also shown in Figure 3C, resulting from two independent experiments (each with forward and reverse conditions). Closed circles: proteins significant in both experiments; open triangles: proteins significant only in forward experiments; open squares: proteins significant only in reverse experiments. TMPO 1-3 are different isoforms of the protein (thymopoietin, LAP2α, LAP2β, LAP2γ).

In RAPIDS, the APEX-moiety is not directly fused to the protein of interest, but rather targeted to it in a rapamycin-dependent manner. Quantitative mass spectrometry then allows a faithful comparison of biotinylated proteins under single-parameter-change conditions (i.e., +/- rapamycin). We recently used RAPIDS for the identification of proteins that are in close proximity to VAPB, a protein residing at the ER and also at the INM [27]. First, we used a plasmid coding for a nuclear fusion protein comprising the peroxidase, two GFP-moieties, a classic nuclear localization signal and the rapamycin interaction domain FKBP12 (APEX2-dGFP-NLS-FKBP12; Figure 3A). As an alternative, we also used a similar reporter lacking the NLS (see below). In HeLa cells co-expressing APEX2-dGFP-NLS-FKBP12 and mCherry-FRB-emerin, the addition of rapamycin resulted in a fast re-distribution of the green reporter protein from the nuclear interior to the nuclear periphery, indicating association with the INM-protein mCherry-FRB-emerin (Figure 3B). The experimental setup is further depicted in Figure 3C. HeLa cells that had been grown in SILAC medium containing either heavy or light isotopes of lysine and arginine were co-transfected with plasmids coding for APEX2-dGFP-NLS-FKBP12 and mCherry-FRB-emerin. Co-transfected cells were incubated with biotin-phenol and rapamycin for 30 min. A short pulse of H_2_O_2_ for one minute then initiated biotinylation of proteins in close proximity to the APEX2-fusion protein. Cells were then lysed and biotinylated proteins were captured using immobilized Neutravidin. As shown in Figure S1, only a few proteins that are biotinylated endogenously were detected when biotin-phenol or H_2_O_2_ were omitted from the reaction or when cells were not transfected with a plasmid coding for APEX2. Addition of H_2_O_2_ led to a strong increase in the observed intensities of biotinylated species, both in the total cell lysates and in the eluted fractions, indicating an APEX2-dependent modification. The overall pattern of biotinylated proteins appeared very similar in rapamycin-treated and non-treated cells, suggesting that a large number of proteins can be modified in an unspecific manner (Figure S1). To specifically analyze proteins that should predominantly be biotinylated upon targeting of APEX2-dGFP-NLS-FKBP12 to mCherry-FRB-emerin, we next probed the Western blots with specific antibodies. Figure 4A shows similar levels of the APEX2-fusion protein in the total cell lysates and, furthermore, similar amounts of self-biotinylated protein as bound to the Neutravidin beads. Biotinylated proteins, as detected by HRP-coupled streptavidin, are detected in Figure 4B. Note that the overall pattern of biotinylated proteins was very similar in the absence or presence of rapamycin, demonstrating that sensitive methods are required to detect specific differences. Indeed, in both forward and reverse reactions, the addition of rapamycin to the cells strongly increased the amount of biotinylated mCherry-FRB-emerin that was eluted from the Neutravidin beads (Figure 4C), indicating successful modification of the rapamycin-dependent binding partner of APEX2-dGFP-NLS-FKBP12. Strikingly, not only this immediate binding partner was detected upon addition of rapamycin, but also the established emerin-binding protein lamin A (Figure 4C, bottom). Endogenous emerin, on the other hand, was not detected as a biotinylated protein in this experiment. This may result from the lower expression level of mCherry-FRB-emerin compared to the endogenous protein (compare Figure 2C, where higher levels of APEX-2-emerin than endogenous emerin were detected). Furthermore, mCherry-FRB-emerin may not readily associate with the endogenous protein, unlike APEX2-emerin.

Based on these results, we decided to subject the samples eluted from the Neutravidin beads to quantitative mass spectrometry. In this approach, cells that were treated with or without rapamycin had initially be grown in media containing either heavy or light amino acids as described above. Hence, a comparison of heavy and light tryptic fragments of biotinylated proteins should immediately yield proteins that were in close proximity to mCherry-FRB-emerin in the presence of rapamycin. Figure 3C (bottom right) and Figure 4D show the combined results of two independent SILAC experiments, with and without rapamycin-induced targeting of APEX2-dGFP-NLS-FKBP12 to the INM-protein mCherry-FRB-emerin and each comprising forward and reverse labeling. For the vast majority of proteins, rapamycin did not lead to a change of the ratio of heavy and light peptides (resulting in a log2-ratio around zero), indicating that the original proteins were either biotinylated endogenously, that is, independently of APEX2, or non-specifically. These proteins are likely to correspond to the equally biotinylated proteins (+/- rapamycin) as detected in the Western blot in Figure 4B. Several proteins, however, were clearly affected by the addition of rapamycin, both in the forward and the reverse reaction, yielding higher levels of biotinylation in the presence of the drug. The most prominent of these proteins, which appear in quadrant IV of the plot, are depicted in Figure 4D. They include emerin itself, probably reflecting modification of the exogenous form of the protein, mCherry-FRB-emerin, lamins B1 and A/C, other proteins of the INM like LAP1 (TOR1AIP1) and LAP2 (TMPO, thymopoietin), nucleoporins Nup153 and Tpr and the ER-protein calnexin. Another prominent hit is MTOR (mammalian target of rapamycin), which probably results from binding of FKBP12-containing APEX2 to the endogenous protein. A list of proteins that were identified in the two independent experiments is presented in Table S2. 

**Figure 5:**
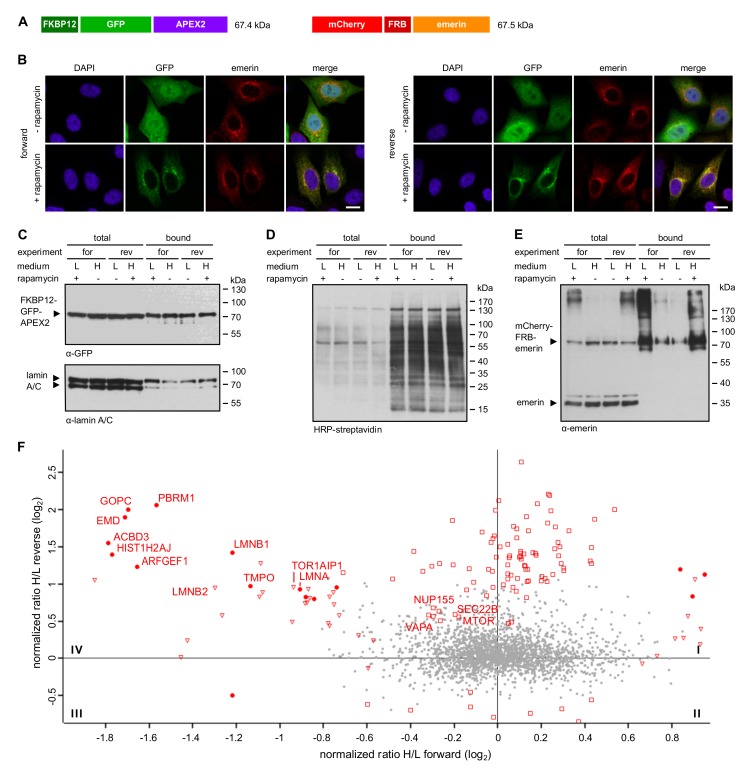
Proximity mapping of emerin by RAPIDS. (**A**) Scheme of FKBP12-GFP-APEX2 and mCherry-FRB-emerin. (**B**–**E**) Cells were co-transfected with plasmids coding for FKBP12-GFP-APEX2 and mCherry-FRB-emerin, grown in “light” (L) or “heavy” (H) medium and treated with or without rapamycin. (**B**) Cells were analyzed by confocal microscopy, detecting the GFP-signal of FKBP12-GFP-APEX2 and the mCherry-signal of mCherry-FRB-emerin. Bars, 10 µm. (**C**–**E**) Cells were subjected to APEX-dependent biotinylation in forward (for) and reverse (rev) reactions (compare Figure 3C for experimental workflow). Proteins from cell lysates were bound to Neutravidin beads and the total and the bound fractions were analyzed by immunoblotting. Anti-GFP and anti-lamin A/C (C), HRP-streptavidin (**D**) or anti-emerin (**E**) were used for detection. Note that the levels of mCherry-FRB-emerin were much lower than those of the endogenous protein. (**F**) The scatter plot resulting from two independent experiments (each with forward and reverse conditions) focuses on quadrant IV and shows normalized log_2_-ratios of proteins eluted from Neutravidin beads in forward (heavy medium (H), without rapamycin; light (L) medium, with rapamycin; x-axis) and reverse (heavy medium (H), with rapamycin; light (L) medium, without rapamycin y-axis) experiments. Closed circles: proteins significant in both experiments; open triangles: proteins significant only in forward experiments; open squares: proteins significant only in reverse experiments.

**Figure 6:**
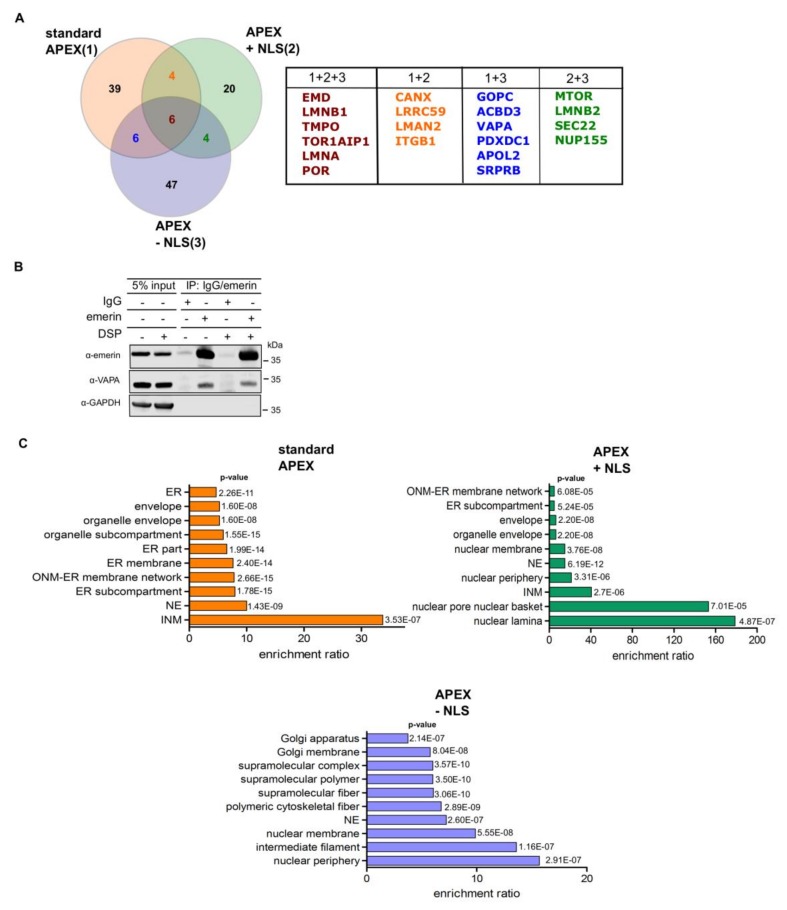
(**A**) Venn-diagram of proteins identified to be in close proximity to emerin in three different approaches: “standard APEX (1)” (see Figure 1 and Figure 2), “APEX plus NLS (2)” (APEX2-dGFP-NLS-FKBP12; see Figure 3, Figure 4) and “APEX minus NLS (3)” (FKBP12-GFP-APEX2; see Figure 5). For the analysis, proteins were included that were identified as significant hits at least once. See also Table S4 for a list of relevant proteins. (**B**) HeLa cells were treated with or without the crosslinking reagent DSP and cellular lysates were subjected to co-immunoprecipitation reactions using mouse anti-emerin antibodies or IgG as a control. Precipitated proteins as well as 5% of the original lysate were analyzed by SDS-PAGE followed by Western blotting. For detection, mouse-anti-emerin, mouse-anti-VAPA and rabbit-anti-GAPDH were used. (**C**) Gene Ontology cellular compartment classification of all significant proteins (Table S4) identified in three different approaches as shown in (**A**).

Based on the design of the APEX2-reporter, the approach described above should favor the identification of nuclear proteins. For a more unbiased approach, we next, performed a similar set of RAPIDS experiments, now with FKBP12-GFP-APEX2 (Figure 5), that is, a version of APEX2 that should not be confined to the nucleus as APEX2-dGFP-NLS-FKBP12, but equally distribute between the nucleus and the cytoplasm. Again, the addition of rapamycin to cells co-expressing FKBP12-GFP-APEX2 and mCherry-FRB-emerin resulted in recruitment of the APEX2-fusion protein to the nuclear envelope and the ER (Figure 5B). Similar transfection rates for FKBP12-GFP-APEX2 are shown in Figure 5C. Western blot analysis of total protein and Neutravidin-bound protein then revealed similar levels of biotinylated proteins under different conditions (Figure 5D) as well as rapamycin-dependent biotinylation of mCherry-FRB-emerin (Figure 5E) and to some extent, of lamins (Figure 5C). As in the previous approach, we did not observe biotinylated endogenous emerin (compare Figure 4C). The results of the proteomic analysis are depicted in Figure 5F (see also Table S3). Again, several known binding partners of emerin or proteins that reside in close proximity to emerin were identified using this approach. A Venn-diagram showing the results for the three types of APEX2-experiments performed (“standard” APEX (Figure 1; Figure 2), APEX with NLS (Figure 3 and Figure 4), APEX without NLS (Figure 5)) is depicted in Figure 6A and relevant proteins are listed in Table S4. 20 proteins were identified in at least two approaches. Next, we performed co-immunoprecipitation experiments to analyze the interaction of endogenous emerin with a potential binding partner. As shown in Figure 6B, endogenous vesicle-associated membrane protein–associated protein A (VAPA), which had been identified in two of the three APEX2-approaches (1 + 3 in Figure 6A), was immunoprecipitated together with emerin when cells had been treated with or without a cross-linking reagent. This result clearly demonstrates that not only overexpressed emerin is in close proximity to VAPA (as detected by RAPIDS), but also the endogenous protein. Finally, we performed gene ontology analyses (Figure 6C). As expected, the standard approach yielded high enrichment ratios for the nuclear and ER-compartments. Similar results were obtained for RAPIDS using the APEX2-construct lacking an NLS. For APEX2 residing largely in the nucleus (APEX2 + NLS) an even stronger focus on nuclear components was observed.

## 4. Discussion

Proximity-dependent biotinylation approaches have been used very successfully for the analysis of the interactome of many proteins [20,21]. The described methods have in common that they all identify protein neighbors and physical interaction has to be investigated by other approaches. Here, we used different versions of APEX2-dependent biotinylation to probe the environment of emerin at the INM and the ER. First, we used the “standard” approach, where APEX2 was directly fused to emerin. Second, we performed two types of RAPIDS, one with a nuclear version of APEX2 and one with a version that resides in the nucleus and the cytoplasm alike. In both cases, the FKBP12-APEX2 interacts with FRB-tagged mCherry-emerin only upon addition of rapamycin to the cells. Quantitative proteomics then allows a faithful identification of real binding partners or neighboring proteins. This becomes obvious when one compares the results of the “standard” APEX-experiment (Figure 2D) and RAPIDS (Figure 4D and Figure 5F). Clearly, the latter yielded a more defined cloud of non-specifically biotinylated proteins around the intersection point of the x- and y-axis in the scatter plot as compared to the more elongated shape of this population in Figure 2D. Thus, a careful design of the experimental conditions in RAPIDS should allow a better discrimination of specific versus unspecific neighbors, compared to other proximity-based methods. 

The identification of proteins that interact directly with our protein of interest, emerin, is not trivial, since for affinity approaches, the conditions that are needed to release proteins from the nuclear lamina may also lead to the disruption of specific protein-protein interactions. Indeed, several reported binding partners of emerin were not identified in a large scale affinity approach [34], including certain nesprins and β-catenin. Using RAPIDS, we identified several known interaction partners of emerin, including LAP1 (TOR1AIP; [35]) and A- and B-type lamins. Lamins are rather abundant proteins with a few million copies per HeLa cell for lamin A and lamin B1 [36]. Interestingly, lamin B2, which is far less abundant than lamin A, was also identified with similar significance levels as lamin A, suggesting a specific interaction. Furthermore, several proteins of the INM were found, e.g. LAP2β (thymopoietin, TMPO). Interestingly, emerin was identified as a proximity partner of LAP2β using BioID-approaches [37]. Emerin itself was also identified in our screen. This could result from biotinylation of mCherry-FRB-emerin and/or endogenous emerin, which is known to interact with itself, at least in vitro [33]. We also found the nucleoporins Tpr, Nup153 and Nup155 in our screen. These seem to be specific proximity partners, because other nucleoporins, whose abundance is expected to be very similar, were not identified. Tpr and Nup153 localize to the nuclear basket of the NPC, that is, in close proximity to the nuclear lamina and emerin. Nup 155 is a component of the inner ring of the NPC. Whether this reflects a direct interaction or a close proximity remains to be investigated in future studies. Interestingly, Tpr and Nup155 were previously identified in emerin-containing complexes [34]. Another potential interaction partner of emerin is VAPA, which was found to coprecipitate with emerin (Figure 6B). Importantly, only endogenous proteins were analyzed in this experiment. VAPA has been described as a protein of the ER [38]. Emerin was previously detected at the level of the ER as well [12] and also suggested to exchange between ER and NE [39]. Furthermore, emerin is initially inserted into the ER-membrane system in a post-translational manner [13]. Hence, it is possible that the two proteins interact at the level of the ER. It remains to be investigated whether VAPA can also reach the INM and whether it is a direct or an indirect interaction partner of emerin.

What is the difference between our three types of SILAC experiments (Figure 6), particularly between RAPIDS using APEX2-constructs containing or lacking an NLS? In principle, the addition of rapamycin to cells is expected to sequester the diffusible FKBP12-containing proteins to all sites where mCherry-FRB-emerin is present. Hence, also the large APEX2-dGFP-NLS-FKBP12 could leave the nucleus and bind to targets at ER-membranes (Figure 3B). The smaller APEX-version, FKBP12-GFP-APEX2, initially localized all over the cell and was then recruited to the ER upon addition of rapamycin in cells co-expressing mCherry-FRB-emerin (Figure 5B). Thus, both proteins should be able to induce biotinylation of INM-proteins as well as ER-membrane proteins facing the cytoplasm. Nevertheless, the spectrum of identified proteins is somewhat different for the two types of APEX2-constructs. Major partners of emerin were identified with both versions (e.g., lamins and LAP1; these were also found in our “standard” APEX-approach, Figure 1 and Figure 2), whereas Tpr and Nup153 were only found with the nuclear version of APEX. APEX2 lacking the NLS, on the other hand, identified a number of additional ER-proteins like ARFGEF1 and VAPA (for comparison see Figure 6 and Table S4). The observed differences may result from the different sizes of the APEX constructs (97.4 vs. 67.4 kDa) and/or their enzymatic domains residing at opposite ends (N-terminal vs. C-terminal) of the proteins. Compared to RAPIDS, the “standard” APEX-approach offers less control over the experimental conditions, since the differentiation between specific and non-specific hits relies on the expression of different proteins (APEX2-emerin versus GFP-APEX2; Figure 1) and subsequent comparison of biotinylated proteins by SILAC. Nevertheless, the method identified several proteins, which were also found in RAPIDS (Figure 6A). Note, however, that the “standard” APEX experiment was only performed once (yet with biological and technical replicates). 

Several reasons could account for the fact that some established binding partners of emerin were not identified in our screens. First, the protein must be expressed in the cell line used for RAPIDS at a sufficiently high level, so that it binds not only to endogenous but also to exogenous emerin. Second, the affinity may not be high enough to allow a faithful detection. The histone deacetylase HDAC3, for example, has a rather low affinity for emerin (7.3 µM; [18]) and may thus escape biotinylation. Third, appropriate sites for biotinylation must be available on the surface of potential proximity partners. Small proteins like BAF (89 amino acids), a protein that binds to the N-terminal region of emerin [9], may lack such sites. They could also be protected by other proteins, impeding modification by the biotin-phenoxyl radicals. Fourth, the tag of our overexpressed version of emerin could affect protein-protein interactions. mCherry-FRB-emerin and APEX2-emerin retain the natural C-terminal end of emerin to allow post-translational ER-insertion as a tail-anchored protein. They could, however, interfere with binding of proteins like BAF to the N-terminal region. Furthermore, the size of the tag could affect the efficiency of targeting of the fusion proteins to the INM [40]. In fact, mCherry-FRB-emerin is about 10 kD larger than APEX2-emerin. Although mCherry-FRB-emerin clearly reached the INM, a substantial portion was observed at the level of the ER (Figure 3B and Figure 5B).

In summary, the choice of tags and their position in the fusion proteins could affect the results of biotinylation-based approaches for the identification of proximity partners. A direct fusion of APEX2 to emerin is straight-forward and yielded meaningful results. Depending on the protein of interest, however, such direct fusions may hamper the identification of certain partner proteins, for example as a result of inefficient targeting to the final destination [27]. Compared to this standard approach, RAPIDS offers the advantage of a physical separation of the radical-generating enzyme and the protein of interest. This allows focusing on specific cellular compartments, for example the nucleus, simply by targeting APEX2 to this organelle, prior to its rapamycin-mediated dimerization with a protein of interest (see Figure 6B). Notably, a similar approach was used very recently in the context of BioID [37]. Furthermore, a simple, single-parameter-change (+/- rapamycin) allows a faithful evaluation of specific versus unspecific hits. One caveat of the method as described here results from possible effects of overexpression of the protein of interest. This could be avoided by (i.) the generation of stable cell lines with options to control the expression level of, for example, mCherry-FRB-emerin and/or (ii.) by depleting endogenous proteins with specific siRNAs. On the other hand, overexpression may allow the identification of proteins, which interact (or are in close proximity) only transiently. One example here could be VAPA, an ER-protein that as such is not expected to interact with the INM-protein emerin. The biological significance of the emerin-VAPA interaction remains to be investigated.

## Supplementary Materials

The following are available online at https://www.mdpi.com/2073-4409/9/3/605/s1. Figure S1: H_2_O_2_- and biotin-phenol specific modification of proteins by APEX2. Cells were transfected with a plasmid coding for mCherry-FRB-emerin and with or without a plasmid coding for APEX2-dGFP-NLS-FKBP12 (+/- APEX2) and subjected to APEX2-dependent biotinylation in the absence (-) or presence (+) of biotin-phenol, rapamycin or H_2_O_2_, as indicated. Proteins from cell lysates were bound to Neutravidin beads and the total and the bound fractions were analyzed by Western blotting. HRP-streptavidin was used for the detection of biotinylated proteins. Proteins detected without expression of APEX2 are endogenously biotinylated, Table S1: Biotinylation assay using APEX2-emerin (standard approach), Table S2: RAPIDS using APEX2-dGFP-NLS-FKBP12 and mCherry-FRB-emerin, Table S3: RAPIDS using FKBP12-GFP-APEX2 and mCherry-FRB-emerin, Table S4: Venn analysis for comparison of all biotinylation experiments targeting emerin, Table S5: Antibodies used in this study.
