# Supplementary material for: Probing the Environment of Emerin by Enhanced Ascorbate Peroxidase 2 (APEX2)-Mediated Proximity Labeling
Source: Cells. 2020 Mar 3;9(3):605. doi: 10.3390/cells9030605 (PMC7140434; doi:10.3390/cells9030605)
Supplement: Supplementary file 1 [file cells-09-00605-s001.zip › tables/Table S5 new.docx]

**Table S5**

**Antibodies**

| **Name** | **Species** | **Origin** | **Application** | **Dilution** |
| --- | --- | --- | --- | --- |
| **Primary antibodies** | | | | |
| α-APEX | rabbit | Peter Rehling, Göttingen, Germany | Western blotting | 1:1,000 |
| α-emerin | rabbit | raised by Genosphere Biotechnologies, Paris, France  antigen: synthetic peptide (C)FPDADAFHHQVHDDDLL  residues 124-140 of human emerin | Western blotting | 1:1,000 |
| α-emerin | rabbit | #10351-1-AP, Proteintech Group, Rosemont, IL, USA | immunofluorescence | 1:1,000 |
| α-emerin | mouse | #AMAb90562, Sigma-Aldrich, St. Louis, MO, USA | immunoprecipitation | 1:1000 |
| α-GFP | rat | #3H9, ChromoTek, Planegg-Martinsried, Germany | Western blotting | 1:1,000 |
| α-GAPDH | rabbit | #10494-1-AP, ProteinTech Group, Rosemont, IL, USA | Western blotting | 1:1000 |
| α-laminA/C | rabbit | #2032, Cell signaling Technology, Danvers, MA, USA | Western blotting | 1:1,000 |
| α-VAPA | mouse | Clone N4 79/12 75-498, Davis/NIH Neuro Mab facility, UC | Western blotting | 1:500 |
| **Secondary antibodies** | | | | |
| α-rabbit-HRP | donkey | #711-035-152, Jackson ImmunoResearch Laboratories, West Grove, PA, USA | Western blotting | 1:10,000 |
| α-rat-HRP | goat | #112-035-003, Jackson ImmunoResearch Laboratories, West Grove, PA, USA | Western blotting | 1:10,000 |
| α-rabbit-Alexa488 | donkey | #A-21206, Molecular Probes, Eugene, OR, USA | immunofluorescence | 1:1,000 |
