# Supplementary figures and images for: Probing the Environment of Emerin by Enhanced Ascorbate Peroxidase 2 (APEX2)-Mediated Proximity Labeling
Source: Cells. 2020 Mar 3;9(3):605. doi: 10.3390/cells9030605 (PMC7140434; doi:10.3390/cells9030605)

Figure S1

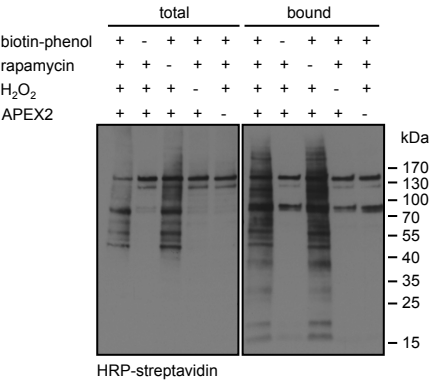

Supplement: Supplementary file 1 [file cells-09-00605-s001.zip › PDFs/figure S1.pdf]
